# Deep Deterministic Policy Gradient-Based Autonomous Driving for Mobile Robots in Sparse Reward Environments

**DOI:** 10.3390/s22249574

**Published:** 2022-12-07

**Authors:** Minjae Park, Seok Young Lee, Jin Seok Hong, Nam Kyu Kwon

**Affiliations:** 1 Department of Electronic Engineering, Yeungnam University, Gyeongsan 38541, Republic of Korea; 2 Department of Electronic Engineering, Soonchunhyang University, Asan 31538, Republic of Korea

**Keywords:** reinforcement learning, deep deterministic policy gradient, hindsight experience replay, mobile robot, autonomous driving, sparse reward environments

## Abstract

In this paper, we propose a deep deterministic policy gradient (DDPG)-based path-planning method for mobile robots by applying the hindsight experience replay (HER) technique to overcome the performance degradation resulting from sparse reward problems occurring in autonomous driving mobile robots. The mobile robot in our analysis was a robot operating system-based TurtleBot3, and the experimental environment was a virtual simulation based on Gazebo. A fully connected neural network was used as the DDPG network based on the actor–critic architecture. Noise was added to the actor network. The robot recognized an unknown environment by measuring distances using a laser sensor and determined the optimized policy to reach its destination. The HER technique improved the learning performance by generating three new episodes with normal experience from a failed episode. The proposed method demonstrated that the HER technique could help mitigate the sparse reward problem; this was further corroborated by the successful autonomous driving results obtained after applying the proposed method to two reward systems, as well as actual experimental results.

## 1. Introduction

Mobile robots are widely used in various fields, such as in industries, hospitals, and the military, and they are trained to perform several activities, including rescuing people [1], transporting heavy objects [2], monitoring, and reconnaissance [3], among other applications [4,5,6]. In general, mobile robots can replace humans in dangerous working environments, such as fires [7] and military environments. In addition, these robots are often tasked with goods deliveries in indoor areas with repetitive movements, such as hospitals [8] and restaurants [9]. Notably, autonomous driving of mobile robots has endowed them with the ability to perform all such activities. However, to appropriately perform these activities, an advanced autonomous driving technology that can respond to the surroundings of robots is required. In general, autonomous driving can be divided into surrounding environment recognition, robot location determination, and destination path planning.

During the recognition process, equipment such as cameras, radar, and light detection and ranging (LiDAR) sensors are typically used to gather information on the surrounding environment. Accordingly, based on such information data, path-planning research has been implemented based on the simultaneous localization and mapping (SLAM) technology, which simultaneously performs two procedures: generation of a map of the surrounding environment and location of the robot [10]. Currently, the SLAM method based on LiDAR sensors is considered to be more accurate for position estimation; however, it has a disadvantage in that LiDAR sensors are expensive. In this regard, applying the SLAM method with cameras or sonar sensors produces a lower accuracy than that with LiDAR but benefits from lower sensor costs. Therefore, to ensure a low cost and improved performance, advanced search algorithms or artificial intelligence approaches are typically used in the path-planning process. Accordingly, several studies have used advanced search algorithms or created maps based on SLAM in path planning by acquiring data from cameras and sonar sensors [11,12].

In general, the path-planning process can be divided into global and local path planning. Of these, global path planning is performed by considering all available information on the surrounding environment, and a robot moves based on the resulting plan. In addition, global path planning is primarily used in static environments such as those containing fixed obstacles. By contrast, local path planning uses only partial information regarding the surrounding environment, such as a dynamic environment with moving obstacles, and controls the movement of a robot based on this information. Because the actual environment can either be dynamic or static, research on search algorithms used for local path planning has attracted considerable attention. The path-planning method was first proposed based on the A* algorithm, which avoids obstacles and searches for the shortest path from a starting point to a destination [13]. Generally, the algorithm determines the optimal path with the shortest distance in a static environment, wherein obstacles do not move. However, in a dynamic environment, the A* algorithm struggles to determine the optimal path, and its performance is affected by the heuristic function. Additionally, searching for the optimal path requires a consideration of time and memory usability; this may include storing a considerable amount of data in a large memory bank to reduce time complexity or using a small amount of memory and waiting for the process to be completed even if more time is required. Owing to these limitations, path planning based on reinforcement learning has received considerable attention from several researchers as a new alternative [14].

A major feature of reinforcement learning is that the learning process is similar to that of humans. That is, an agent can navigate complex and difficult situations, sometimes even better than humans. For example, Go [15] and StarCraft [16] represent case studies wherein agents demonstrated better performance than humans based on reinforcement learning. In particular, AlphaGo won a Go match against a human representative in 2016 with a set score of 4:1. Furthermore, MuZero [17], developed by Deep Mind, has demonstrated excellent performance in games such as Go, chess, and shogi, even without game information. Reinforcement learning is primarily used as an algorithm to easily cope with unexpected situations. In addition, path planning based on reinforcement learning does not necessitate the storage of a large amount of data and can immediately use the data received in real time. In this case, even if a new obstacle is added or if the environment changes, mobile robots can search for the optimal path. Thus, reinforcement learning is a type of machine learning approach that can determine the optimal policy via a reward mechanism and perform actions based on information from the environment as observed by the agent. Therefore, reinforcement learning is considered suitable for the path planning of mobile robots.

Generally, reinforcement learning can be divided into value- and policy-based learning. Of these, value-based learning is used to handle a discrete action space and refers to the identification of the optimal policy that maximizes the cumulative reward using a value function that evaluates a state or action. A representative algorithm here is the deep Q network (DQN), which combines Q-learning and an artificial neural network that approximates the value function and describes the process of developing policies to successfully play Atari games [18]. However, policy-based learning does not use an artificial neural network as the value function but directly approximates a policy that maximizes the cumulative reward. Therefore, policy-based learning can handle continuous action spaces [19]. Notably, policy gradient, a policy-based learning algorithm, is widely used in the field of robotics owing to the need to handle continuous action spaces.

Policy gradient-based algorithms are typically developed using the REINFORCE [20] and actor–critic [21] methods. Both these algorithms involve an approximation of some policy via an artificial neural network; however, the difference between the two is that REINFORCE updates the policy after the episode terminates, whereas actor–critic updates the policy at every step. A representative policy-based algorithm that uses the actor–critic framework is the deep deterministic policy gradient (DDPG) [22], which combines the deterministic policy gradient (DPG) [23] and DQN. In particular, two primary ideas of DQN, experience replay and target network separation, are borrowed to ensure learning stability. Recently, a study wherein a mobile robot did not collide with obstacles and used DDPG to search for destinations in an environment without obstacles, an environment with four fixed obstacles, and a maze with two moving obstacles was reported [24]. The foregoing study [24] used a state consisting of 10 values obtained from the laser distance sensor placed at 180° in front of the robot, the action of the previous state, and the distance to the destination, and it used an action consisting of linear and angular velocities. The reported results [24] revealed that the agent could easily determine the optimal policy in environments without obstacles, but it struggled in complex environments with obstacles. This implies that this reward system is ineffective in more complex cases with obstacles. Another study [25] involved path planning for mobile robots in various environments using a dueling DDPG architecture and a dense reward system. The results [25] revealed that the adopted method was effective in complex environments such as small offices and warehouses, which are typical. The results reported in [24,25] demonstrated that designing a dense reward system suitable for each complex environment is often difficult. Another study [26] implemented path planning for mobile robots in various environments using Mixed Noise-LSTM-DDPG (MN-LSTM-DDPG). The results [26] indicated improved performance compared with other existing algorithms [24,27,28]; note that the authors of the foregoing study did not apply the hindsight experience replay (HER) [29] technique but proposed only a technical methodology based on DDPG. Based on these results, we can conclude that an advanced reward system or reinforcement learning algorithm must be applied to achieve successful autonomous driving in a complex environment. That is, these technologies can be applied for successful learning by solving the sparse reward problem, which stands out in a complex environment. However, the implementation of these technologies is difficult without sufficient expertise and experience. In addition, setting the optimal reward system based on trial and error considerably increases costs. In particular, in the path planning for robot navigation, the number of times a goal is achieved through exploration using random actions is significantly low; therefore, the probability of encountering a sparse reward system is high. In addition, these observations indicate that, apart from the problems of well-designed reward engineering, finding the optimal policy is more difficult due to the lack of fundamental experience in achieving the goal. Furthermore, the number of studies to apply the HER to solve this problem is in sufficient. The contribution of this study is that HER was used to verify the feasibility of solving the sparse reward problem encountered in the autonomous driving of mobile robots.

Previously, the HER technique has been used to reevaluate the database in a buffer memory to generate normal experiences. In particular, for a failure episode, new normal trajectories are regenerated based on this episode as follows: (1) a random point of the failure episode is selected as the new destination, (2) the trajectory is regenerated from the beginning of the episode to the new destination, and (3) data transition of the new trajectory is added to the replay buffer memory. Based on this process, the sparse reward problem caused by the lack of fundamental experience in achieving goals can be alleviated, and normal data can be sampled efficiently. Consequently, the optimal policy can be determined without advanced reward engineering. Because entirely setting the reward system is often difficult, the HER technique can assist reward engineering in searching for the optimal policy.

With this background, herein, we propose a method for solving the sparse reward problem occurring in the path planning of autonomous driving mobile robots while searching for the optimal policy using the DDPG and HER techniques. The proposed method is executed on reward systems divided into two cases and implemented in the robot operating system (ROS)-based TurtleBot3 Burger in the Gazebo simulation. The states used in the experiment consist of the laser distance sensor values, previous action, and distances to the destination, and the actions consist of linear and angular velocities. Subsequently, we implement DDPG networks as the policy and value networks, and the HER technique is used as follows: three arbitrary coordinates are selected as the new destinations for a trajectory that failed to achieve the goal, and three trajectories are created using the new destinations as normal experiences. To demonstrate the effectiveness of the proposed method, we compare the results of each HER application experiment in a simulation and implement navigation in a real-world environment.

The experimental results reveal that applying the HER technique can help alleviate the sparse reward problem encountered in the autonomous driving of mobile robots and help determine the optimal policy. The primary contributions of this study can be summarized as follows:

- A DDPG-based application method of the HER technique for solving the sparse reward problem encountered in the autonomous driving of mobile robots is introduced.
- The proposed HER application method demonstrates the possibility of solving the sparse reward problem of the simplest reward system in the autonomous driving of mobile robots.
- The proposed HER application method can be applied with reward engineering to improve the autonomous driving performance of mobile robots, and this method can be used instead of creating a perfect reward system.

## 2. Related Research

Notably, in the robotics field, artificial intelligence-based research for path planning to facilitate completely autonomous driving of mobile robots is gradually progressing, and studies have been conducted based on reinforcement learning. In this regard, a study was conducted based on value-based learning by implementing path planning, and using this, the robot avoided obstacles in indoor simulation environments by combining Q-learning with an artificial neural network [30]. By predicting the Q value using an artificial neural network and training the network using data transitions generated during navigation, the robot succeeded in moving on its own without colliding with obstacles. Similarly, another study was conducted using dueling double DQN to plan a path without colliding with the destination [31]. In detail, here, similar to the method applied for the Atari game with DQN, the surrounding environment was recognized in the form of depth images that were used as the states, and actions were predicted using an artificial neural network. Moreover, the dueling architecture contains more information than the original DQN in the training process owing to the advantage term, and the double DQN can eliminate noise causing an overestimation of the action value. The results reported in [31] demonstrated that the robot could successfully drive autonomously in both simple and complex environments, depending on the number of obstacles encountered in the simulated indoor space. As demonstrated by the foregoing studies, the limitation of value-based learning is that continuous values, such as the velocity and angle, are required to be sampled as discrete values. To overcome this limitation, research has been conducted on path planning based on DDPG, such as that in [32], which can handle continuous action spaces. In the foregoing study, DDPG was used to control the position of a mobile robot. The authors [32] compared the DDPG, DQN, Villela, and IPC methods as position control technologies for mobile robots and determined that DDPG was the fastest and most efficient algorithm to search for the optimal policy for path planning to a destination in environments with and without obstacles. Another study used improved DDPG for the path planning of mobile robots; here, the learning of DDPG was accelerated using a small amount of prior knowledge, thereby improving the performance in determining the optimized policy [33].

In this study, we jointly used the DDPG and HER techniques for the autonomous driving of a mobile robot. Note that a previous study [24] searched for the optimized policy for path planning using DDPG and appropriate reward engineering in a mobile robot simulation environment. The foregoing study [24] was designed to compensate for the change in the distance between the current position of the robot and destination. Here, by contrast, instead of focusing on reward engineering, by applying the HER technique, we test whether the optimized policy for path planning can be determined even if reward engineering is insufficient; consequently, the effort required to design advanced reward engineering is reduced.

## 3. Background

### 3.1. Deep Deterministic Policy Gradient

Over the years, value-based learning has achieved outstanding results in areas that handle discrete action spaces, such as a DQN. However, applying value-based learning to handle a continuous action space is less efficient than the optimization process in the discrete action space because it uses sampled actions to cover the continuous action space. Therefore, studies on policy-based learning have been conducted recently, as policy-based learning can directly handle continuous spaces in fields that require continuous action spaces. Correspondingly, policy-based algorithms have been studied according to the policy gradient, which approximates the policy function as an artificial neural network. The policy gradient determines the policy $\pi_{\theta }$ that maximizes the objective function J(θ) using the gradient ascent method, and the gradient of the objective function (1) is as follows:

(1) $$\nabla_{\theta }J\left(\theta \right){=E}_{\pi_{\theta }}\left[\nabla_{\theta }{\log\pi }_{\theta }\left(s,\;a\right)Q_{\pi_{\theta }}\left(s,\;a\right)\right].$$

Generally, the policy gradient, as an analytical method, is divided into REINFORCE and actor–critic algorithms. Here, first, REINFORCE uses the return $G_{t}$ instead of the Q value $Q_{\pi_{\theta }}\left(s,\;a\right)$ and computes the gradient after the episode ends. Second, the actor–critic uses the output $Q_{w}\left(s,\;a\right)$ of the artificial neural network that approximates the value function instead of $Q_{\pi_{\theta }}\left(s,\;a\right)$ and computes the gradient at every step. Notably, DPG uses deterministic and parameterized actions rather than stochastic policies, and the applications of policy-gradient methods are possible. In addition, parameterized actions reduce the computational complexity associated with the calculation process compared with other algorithms using stochastic policies. However, parameterized actions tend to attempt exploitation more than exploration, resulting in a potential lack of information regarding unknown states. This problem can be mitigated by implementing an off-policy technique that uses stochastic behavior and deterministic target policies. DDPG combines DQN with DPG and searches for the optimal policy using the actor–critic framework in a continuous space. Additionally, Ornstein–Uhlenbeck (OU) noise [34] is added to facilitate exploration.

### 3.2. Hindsight Experience Replay

In reinforcement learning, controlling the balance between exploration and exploitation is crucial for the agent to search for the optimal policy. In particular, this problem is important in environments with sparse reward problems, for example, when a robot struggles to find its destination owing to the lack of normal experience. To solve this problem, the HER technique reevaluates the trajectory part that fails to achieve the goal. That is, the learning is improved by increasing the number of normal episodes by recreating the trajectory that achieves the goal based on the failed trajectory part. For the autonomous driving of mobile robots, because both states and actions belong to the continuous space, reaching the destination solely based on random actions is rare. In this regard, prior knowledge-based reward engineering can be applied to solve this sparse reward problem; however, as the problem becomes more complex, addressing it becomes difficult. Meanwhile, the HER technique helps solve the sparse reward problem without such a reward engineering approach and is easy to combine with other off-policy algorithms, such as DDPG.

## 4. Experiment

### 4.1. Robot Operating System & Gazebo

Notably, the ROS is a software platform used for developing robot applications and is a meta operating system that can be used on Linux, Windows, and Android, which are traditional operating systems. In general, the ROS provides various development and debugging tools necessary for the implementation and development of functions, such as hardware abstraction, sub-device control, sensing based on various sensors, map creation, and motion planning, which are required for robot application programs. In addition, by supporting data transmission and reception between different types of devices, data can be transported between various operating systems, hardware, and programs, enabling the development of robots with several types of hardware.

**Figure 1:**
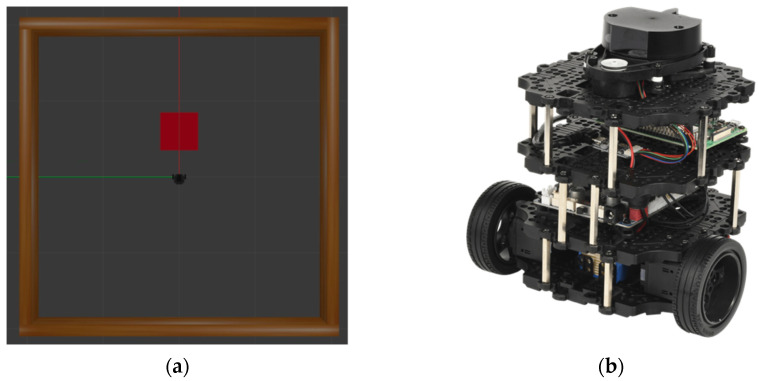
(**a**) Turtlebot3 in the simulation environment. The red square box is the destination, the red line is the *x*-axis, and the green line is the *y*-axis. and (**b**) a real TurtleBot3.

Communication is generally divided into three categories: topic, service, and action. Specifically, topic communication represents a one-way message transmission and reception method, service communication denotes a two-way type of message request and response method, and action communication is a two-way type of message feedback method based on the goal and result. Based on the aforementioned ROS meta operating system, this study identified optimized behavior based on sensor values of a mobile robot in the Gazebo environment and the DDPG algorithm using topic communication between nodes. As depicted in Figure 1a, Gazebo is a three-dimensional (3D) simulator that simulates a physics engine, various sensors, and an experimental environment to simulate reality for robot development. Because Gazebo can be configured to be similar to a real environment, it is widely used as it reduces the time and cost required in actual practice and increases the convenience of development. In addition, it has good compatibility with the ROS. The mobile robot used in this study was TurtleBot, a representative mobile robot based on the ROS, and the detailed model was the TurtleBot3 Burger shown in Figure 1b. Notably, the robot has two Dynamixels on the left and right, which transmit power to the two wheels, and OpenCR is used as the intermediate controller to control them. In addition, a Raspberry Pi 3b+ board and laser distance sensor are used; each of these are operated with the ROS, and the laser is used to measure a distance around the robot in full 360° to comprehend the surrounding environments.

### 4.2. Environment Implementation

The objective of our investigation was to allow the agent to reach the destination without colliding with a wall. The activity area of the robot was a closed square space, and the destination was an arbitrary point within the space marked with a red square. The motion of the robot comprised linear and rotational motions, and the external input for each motion type corresponded to the state of the experiment. One step involved determining the optimal values for the linear and rotational motions in the current state according to the policy and planning movements. The episode ended with three cases: reaching the destination, colliding with a wall, and a timeout situation wherein the agent wandered for the maximum number of action steps without colliding. At the beginning of the episode, the robot’s position was set at the center of a rectangular space bounded by four walls, and the new goal was set at an arbitrary location; following this, the robot performed the action. Exceptionally, if the robot reached its destination, a new episode began at that location. This series of processes was repeated after the episode terminated.

### 4.3. Designing States, Actions, and the Reward System

An action $a_{t}\;\in {\;R}^{2}$, a state $s_{t}\;\in {\;R}^{14}$, and the reward system can be defined as follows, respectively:

(2) $$a_{t}=\left[v_{t},\;\omega_{t}\right]+N,$$

(3) $$0\;\leq {\;v}_{t}\;\leq \;0.22,\;-2\;\leq {\;\omega }_{t}\;\leq \;2,$$

(4) $$s_{t}=\left(l_{t}{,\;d}_{t},\;\phi_{t}{,\;a}_{t-1}\right).$$

**Figure 2:**
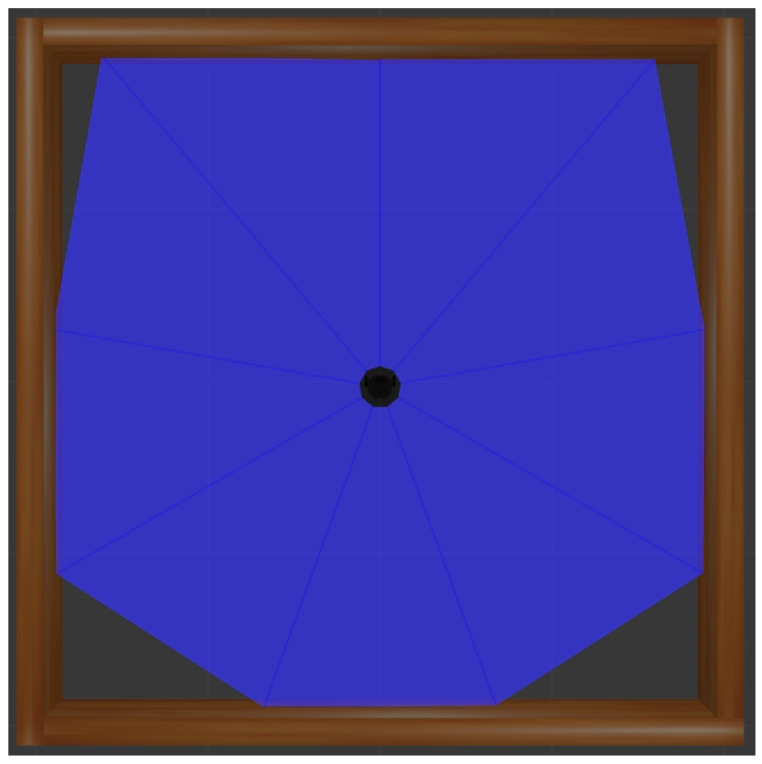
Laser detection range used by the robot to recognize its surroundings.

In (2), the action consists of the linear velocity $v_{t}$ and angular velocity $\omega_{t}$, and OU noise N was added to the action to facilitate exploration. Note that OU noise adds randomness to the action, which is influenced by previous behaviors rather than general randomness. That is, it smoothly changes the speed, which is the action of the mobile robot, according to the change in time, considering the speed of the previous action. In addition, the maximum and minimum values for each velocity are limited, as shown in (3). $l_{t}\;\in {\;R}^{10}$ denotes a vector, including the laser distance sensor (LDS) value, and as shown in Figure 2, the TurtleBot3 Burger uses 10 distance values obtained using the laser sensor at an interval of 36° at approximately 360°. $d_{t}$ denotes the straight-line distance between the coordinates of the goal $P^{g}=\left(P_{x}^{\;g}{,\;P}_{y}^{\;g}\right)$ and current coordinates of the robot $P=\left(P_{x}{,\;P}_{y}\right),$ and it is defined as follows:

(5) $$d_{t}=\sqrt[{}]{{{(P}_{x}^{\;g}\;{-\;P}_{x})}^{2}{+(P}_{y}^{\;g}\;{-\;P}_{y}{)}^{2}}.$$

where $\phi_{t}$ is the difference between the angle of the goal-current position and yaw value, and it is defined as follows:

(6) $$\phi_{t}{=\tan}^{-1}\frac{\left(P_{y}^{\;g}\;{-\;P}_{y}\right)}{\left(P_{x}^{\;g}\;{-\;P}_{x}\right)}\;-\;\phi_{\mathrm{yaw}}.$$

As $\phi_{t}$ approaches zero, the robot moves along the correct direction. Finally, the reward system is defined in three ways according to the experiment. First, when insufficient reward engineering $R_{1}$ is applied, the agent receives a reward of −1 for each step, +1000 upon reaching the goal, and −200 if it collides with the wall:

(7) $$R_{1}\left(s_{t}{,\;a}_{t}{,\;P}^{\;g}\right)=\{\begin{array}{cc} 1000, & {\;\mathrm{if}\;d}_{t}\;<\;0.15\\ -200, & \;\mathrm{if}\;\mathrm{collision}\;\\ -1, & \mathrm{otherwise} \end{array}.$$

Second, in (8), the reward system $R_{2}$ is applied with sufficient reward engineering. In this case, the agent receives a reward of +500 for reaching the destination and −550 for hitting a wall. In addition, the reward is set proportional to the straight-line distance between the goal and the robot’s current location if the distance is greater than zero. By assigning a reward proportional to the distance from the destination, clear information regarding the destination can be provided. Otherwise, the reward is set to −8, as follows:

(8) $${\;R}_{2}\left(s_{t}{,\;a}_{t}{,\;P}^{\;g}\right)=\{\begin{array}{cc} 500, & {\;\mathrm{if}\;d}_{t}\;<\;0.15\\ -550, & \;\mathrm{if}\;\mathrm{collision}\;\\ 200\;\left(d_{t-1}\;{-\;d}_{t}\right), & {\;\mathrm{if}\;(d}_{t-1}\;{-\;d}_{t})\;>\;0\;\\ -8 & \;\mathrm{if}\;\left(d_{t-1}\;{-\;d}_{t}\right)\;\leq \;0 \end{array}.$$

### 4.4. Network Structure

**Figure 3:**
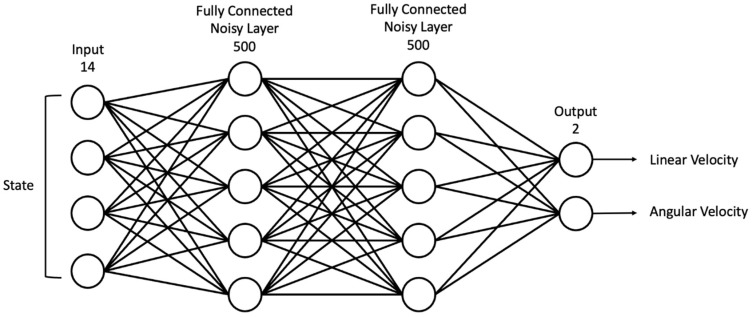
Actor network structure consisting of fully connected layers with noise.

**Figure 4:**
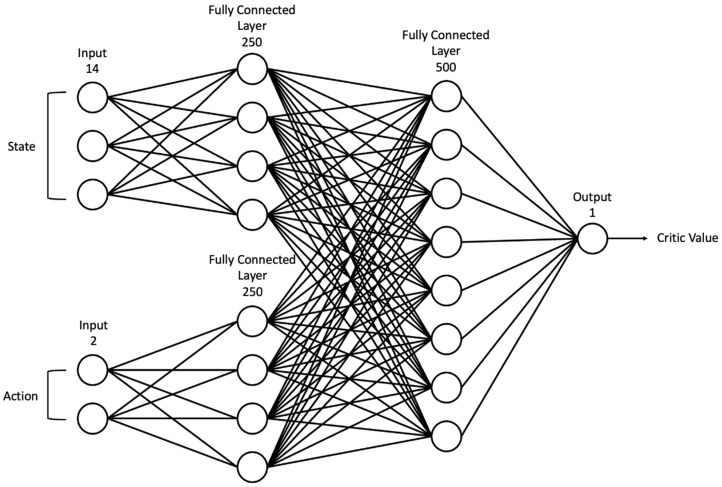
Critic network structure consisting of fully connected layers.

In our study, artificial neural networks were used to approximate actors to select actions for a given state and critique given states and actions. For learning stability, target networks were additionally used for both neural networks. As illustrated in Figure 3, an actor neural network was constructed based on a multilayer perceptron (MLP) architecture with noise. All layers of the actor network were composed of a fully connected layer, and the input layer consisted of 14 nodes and received the current state $s_{t}$ as the input. The hidden layer consisted of 500 nodes with noise at all weights and used a rectified linear unit (ReLU) activation function. The output layer consisted of two nodes, and both nodes denoted a deterministic action $a_{t}$ composed of $v_{t}$ and $\omega_{t}$ using sigmoid and hyperbolic tangent activation functions, respectively. As depicted in Figure 4, the critic network also consisted of an MLP structure and fully connected layer without noise. To evaluate the given state and action pair, the input layer had 14 nodes for the current state $s_{t}$ and two nodes for the action $a_{t}$, which is the result of the actor network. In the first hidden layer, the nodes representing the state and action were connected to 250 nodes each, and all nodes were fully connected to each other in the second hidden layer. The output layer consisted of one node and was designed to provide critique values without an activation function. The actor network was optimized by maximizing the sum of the critique values $Q\left(s_{i}{,\;\mu }_{\theta_{\mu }}\left(s_{i}\right){;\;\theta }_{Q}\right)$ using gradient descent as the loss function, which is defined as follows:

(9) $$L_{i}^{a}=-\underset{i}{\sum }Q\left(s_{i}{,\;\mu }_{\theta_{\mu }}\left(s_{i}\right){;\;\theta }_{Q}\right),$$

where $\mu_{\theta_{\mu }}$ denotes the deterministic policy, and $\theta_{Q}{\;\mathrm{and}\;\theta }_{\mu }$ denote the weights of the critic and actor networks, respectively. In addition, the critic network was optimized to reduce the difference between label $y_{i}$ and critique value $Q\left(s_{i}{,\;a}_{i}{;\;\theta }_{Q}\right)$ using gradient descent with a smooth L1 loss, defined as follows:

(10) $$L_{i}^{c}=\left|Q\left(s_{i}{,\;a}_{i}{;\;\theta }_{Q}\right){\;-\;y}_{i}\right|,$$

where $y_{i}{=r}_{i}+\gamma Q\left(s_{i+1}{,\;\pi }_{\theta_{\mu }^{-}}\left(s_{i+1}\right){;\;\theta }_{Q}^{-}\right)$, γ is a discounting factor, and $\theta_{\mu }^{-}$ and $\theta_{Q}^{-}$ denote the weights of the target actor and critic networks, respectively.

### 4.5. Database Collection Based on HER

**Figure 5:**
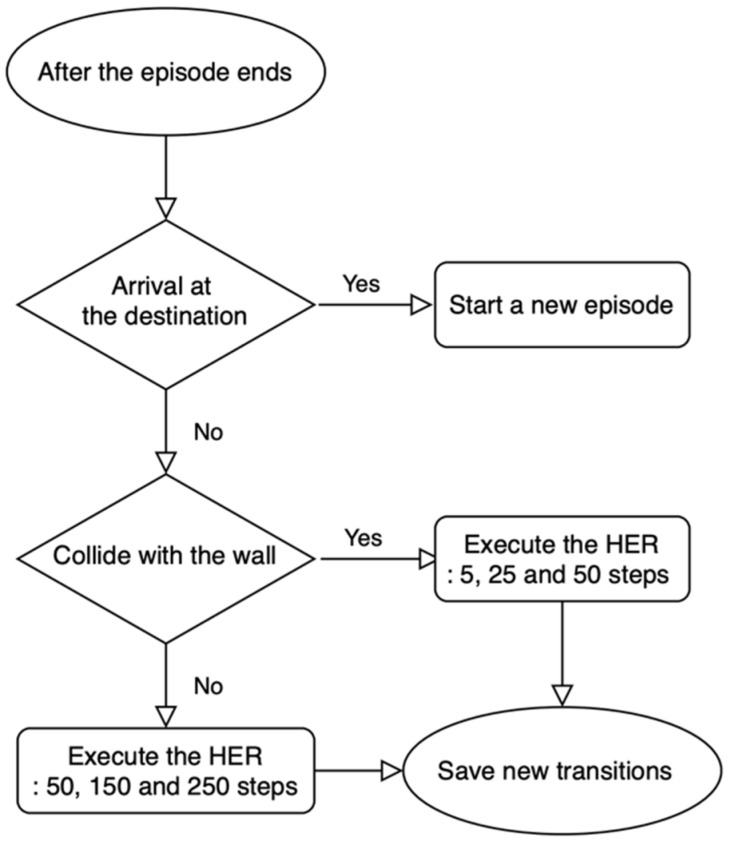
Flowchart for generating new data transitions using the hindsight experience replay (HER) technique.

**Figure 6:**
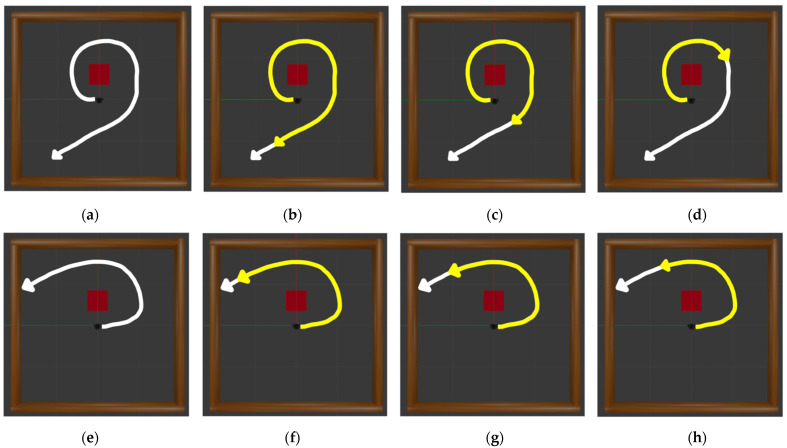
White lines are the trajectories of failed episodes and yellow lines are the sub trajectories generated from these white lines. (**a**) Case of the maximum-step trajectory, and the (**b**–**d**) trajectory generated according to each step of 50, 150, and 250. (**e**) Case of the collision trajectory, and the (**f**–**h**) trajectory generated according to each step of 5, 25, and 50. While the previous reinforcement learning algorithms without HER stores only (**a**,**e**) in the replay buffer, the proposed method appends (**b**–**d**,**f**–**h**) as well as (**a**,**e**), to the replay buffer.

Figure 5 illustrates the overall process of generating new data transitions using the HER technique. Note that the data transition $\left(s_{t}{,\;a}_{t}{,\;r}_{t+1\;}{,\;s}_{t+1}\right)$ used for training is generated when the agent proceeds in one step and is stored in the buffer memory at every step. In general, data transitions are created by exploring random actions until a certain number of data transitions accumulate in the buffer memory. Based on these random actions, the probability of arriving at the destination in a continuous space is low. If the reward function is simple, this converts into a sparse reward problem. In an environment with sparse rewards, the agent cannot be guaranteed to converge to the optimized policy, and the agent may require a long time to learn to converge to the optimized policy. To solve this problem, previous studies have designed more complex reward functions, for instance, by using the distance between the mobile robot and destination as an argument in the reward function. By contrast, we used the HER technique to rapidly collect data transitions that help reinforcement learning, even if the reward function is simple. Algorithm 1 presents the overall process of generating new data transitions using the HER technique. First, when an episode fails, new data transitions are created based on this failed episode. As depicted in Figure 6, when the robot does not reach the destination, three random states are selected within this trajectory as new destinations, and the new trajectories from the beginning of the episode to the new destinations are created and then appended to the buffer memory. For example, Figure 6a presents a failure episode that does not achieve the goal within the maximum number of steps. In Figure 6b–d, the new destinations are selected at 250, 150, and 50 steps before the terminal state, and the data transitions are created from the beginning of the episode to the new destination. Figure 6e presents an episode that ended when the robot collided with a wall. As illustrated in Figure 6f–h, if the robot collides after 50 steps, the new destinations are selected at 50, 25, and 5 steps before the terminal state, and the new data transitions are created and stacked in the buffer memory. Through this process, the HER technique creates the possibility of convergence in situations wherein convergence cannot be achieved owing to the lack of normal experience, and it can simultaneously play a role in creating the possibility of faster convergence.
**Algorithm 1:** Hindsight Experience Replay1: T ← The last step of the episode2: G ← ∅3: if $s_{T}$ is collision4:   G ← $\left\{s_{T\;-\;5}{,\;s}_{T\;-\;25}{,\;s}_{T\;-\;50}\right\}$5: if $s_{T}$ is not goal6:   G $\leftarrow \left\{s_{T\;-\;50}{,\;s}_{T\;-\;150}{,\;s}_{T\;-\;250}\right\}$7: for $g^{\prime }\;\in \;G$ do8:   for t=0, T do9:      $r^{\prime }\;≔\;R\left(s_{t}{,\;a}_{t}{,\;P}^{\;g}{}^{\prime }\right)$10:      if $r^{\prime }$ is goal11:        Break12:   Store the transition $\left(s_{t}g^{\prime }{,\;a}_{t}{,\;r}^{\prime }{,\;s}_{t+1}g^{\prime }\right)$      » || denotes concatenation13:   end for14: end for

## 5. Experimental Results

**Figure 7:**
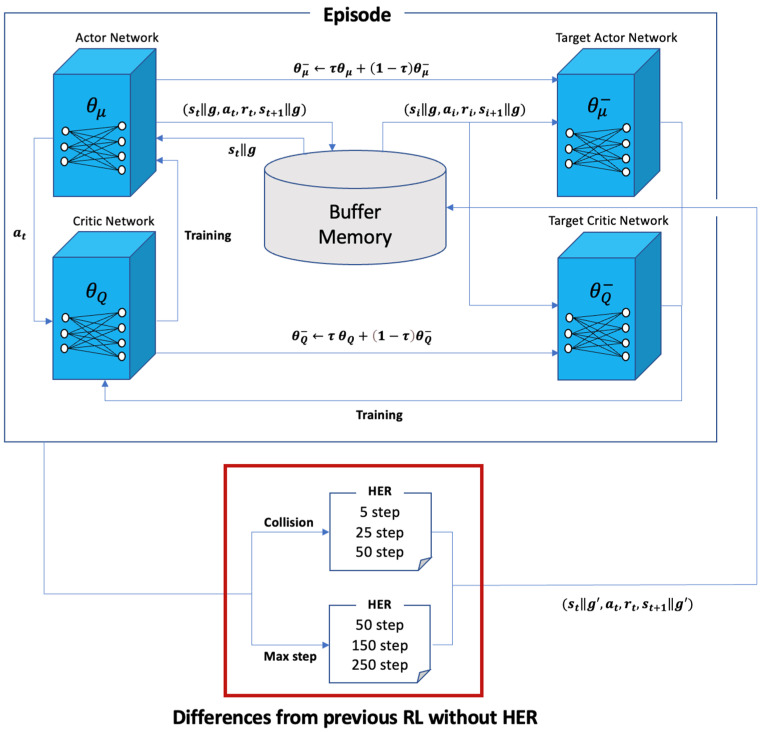
Overall system illustration of reinforcement learning based on deep deterministic policy gradient (DDPG) with HER.

Figure 7 presents the overall system configuration. In one episode, agent TurtleBot3 computes action $a_{t}$ using the randomly initialized actor network and multiplies it by the maximum linear and angular velocities to fit the range of TurtleBot3. Based on this action, after this step, the data transition ${(s}_{t}{,\;a}_{t}{,\;r}_{t+1}{,\;s}_{t+1})$ is stored in the buffer memory. If the length of the buffer memory exceeds the maximum buffer length, the initial data are deleted to maintain the length of the buffer memory. When the minimum buffer length is exceeded, the critic and actor networks are trained at every step using the data transitions sampled according to the batch size from the buffer memory. After the training, the networks are soft updated such that the weights of the critic and actor networks are copied at a certain ratio τ to the target critic and actor networks using (11) and (12).

(11) $$\theta_{Q}^{-}\;\leftarrow {\;\tau \theta }_{Q}+\left(1\;-\;\tau \right)\theta_{Q}^{-},$$

(12) $$\theta_{\mu }^{-}\;\leftarrow {\;\tau \theta }_{\mu }+\left(1\;-\;\tau \right)\theta_{\mu }^{-}.$$

At the end of an episode, HER is applied depending on whether the goal is achieved, and a new data transition is stored in the buffer memory. Further, to demonstrate the effectiveness of the proposed method, three experiments were conducted. Each experiment ran up to 1000 episodes once, and we compared the results of three repeated trials. The episode reward of the training process was used to compare the results, and the average value of the reward for the last 10 episodes was displayed in a graph to emphasize the policy tendency of the optimization process.

The resulting graph consists of two graphs for each experiment. One shows the reward of each episode for each trial in black, yellow, and red, and the other shows the range between the maximum and minimum in gray, and the average of each episode for all three trials is denoted by the blue line.

**Figure 8:**
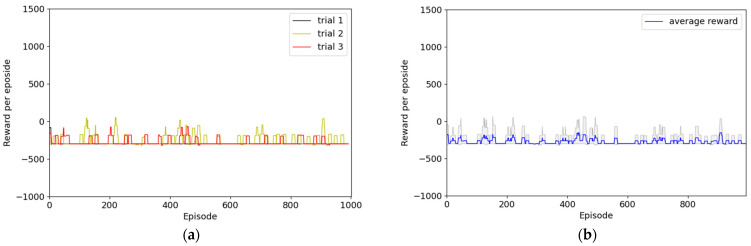
Results of Experiment 1: simplest reward system without HER. (**a**) Episode rewards for each of the three trials; (**b**) maximum, minimum, and average values of episode rewards considering all three episodes.

**Figure 9:**
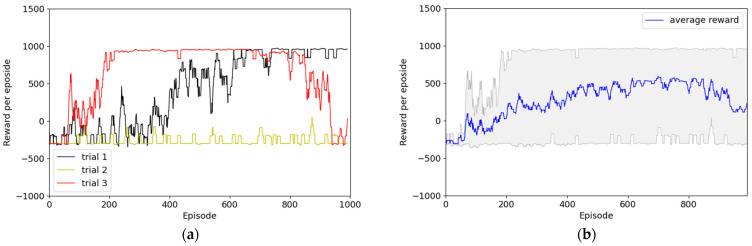
Results of Experiment 1: simplest reward system with HER. (**a**) Episode rewards for each of the three trials; (**b**) maximum, minimum, and average values of episode rewards considering all three episodes.

Experiment 1 used the simplest reward function, as depicted in Figure 8, which represents the result of not applying HER. In all three trials, the average reward of the episode converged to approximately −300 points, and the robot did not reach the destination successfully. The agent was observed to follow the policy of maximizing the rewards to finish the episode without colliding. That is, this score denoted the act of moving around the same space, and we believe that the agent considered completing the episode without colliding while optimizing the policy. In addition to the insufficient experience of the agent in reaching the goal using random search, it had insufficient information to achieve the goal because the reward system lacked reward engineering. However, as presented in Figure 9, although the convergence speeds differed, two out of three episodes converged to +1000 points. This result was obtained by adding the normal experience created by the HER technique; thus, the HER technique can help determine the optimized policy. However, one of the two convergence trials is confirmed to drop to −300 points at approximately 800 episodes. After convergence, the optimized policy was maintained to successfully reach the destination; however, the policy changed rapidly owing to unknown reasons. Thus, the agent could not achieve the goal.

**Figure 10:**
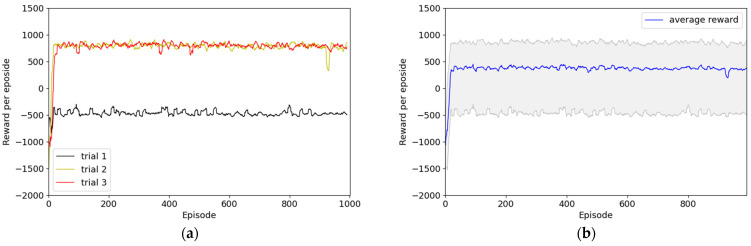
Results of Experiment 2: reward system applied with sufficient reward engineering without HER. (**a**) Episode rewards for each of the three trials; (**b**) maximum, minimum, and average values of episode rewards considering all three episodes.

**Figure 11:**
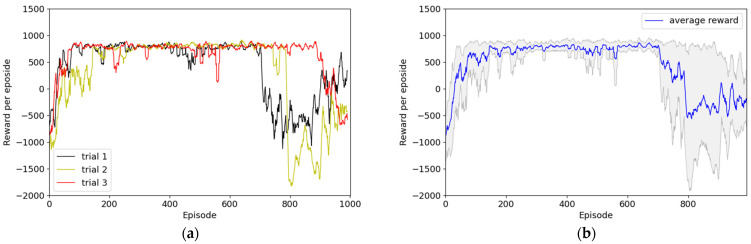
Results of Experiment 2: reward system applied with sufficient reward engineering with HER. (**a**) Episode rewards for each of the three trials; (**b**) maximum, minimum, and average values of episode rewards considering all three episodes.

Second, Experiment 2 consisted of an environment wherein reward engineering was sufficiently applied, and Figure 10 illustrates the reward graph of the results without the HER technique. In one of the three experiments, the reward for most episodes was approximately −500, which indicates that no optimal policy was determined. However, the other two experiments demonstrated convergence to approximately +800 points in approximately 20 episodes, and this may indicate the establishment of a policy to successfully perform path planning to the destination. Figure 11 presents the results obtained with the application of the HER technique. Although the convergence speed differed across all three trials, the convergence was approximately +1000 points within 200 episodes. However, the first trial presented significantly lower reward results for 700 episodes, the second trial for 800 episodes, and the third trial for 900 episodes.

**Figure 12:**
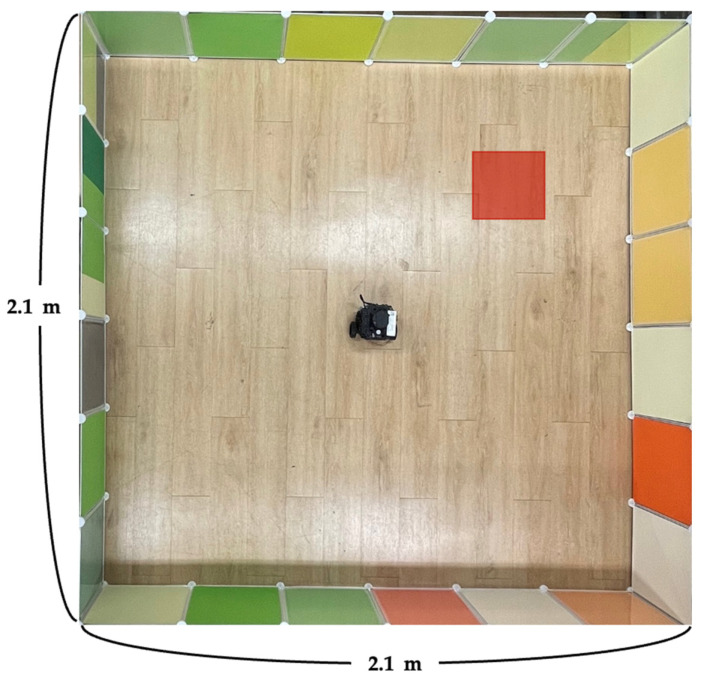
Real-world environment used to verify the proposed method.

Finally, the results of real-world experiments will be discussed. To verify the results in a real-world environment, an experimental environment similar to the simulation environment was established, as presented in Figure 12. To confirm the applicability of the policy learned in the virtual environment in a real environment, each policy obtained by the DDPG algorithm in Experiments 1 and 2 was applied to the real environment. The results of both experiments indicated successful navigation to the destination without difficulty in 10 out of 10 trials. Autonomous driving was accomplished in all attempts; however, this aspect must be considered as learning is assumed to stop after the establishment of the optimal policy.

## 6. Discussion

We demonstrated the potential of the HER technique in solving problems encountered during the autonomous driving of mobile robots through two reward systems and real-world experiments. First, in the reward system with the sparse reward problem, DDPG without HER failed to determine the optimal policy; however, the proposed method aided in determining the optimal policy based on the convergence of the episode reward to a positive value. Second, for a dense reward system, the proposed method produced a higher learning success rate than DDPG without HER, indicating that the proposed method could help determine the optimal policy. Based on these results, the proposed method was confirmed to have a positive effect on learning for both dense and sparse reward systems. In addition, the optimal policy obtained in the virtual environment was applied to a real environment, and the results indicated that it was applicable to the real environment. This result also demonstrates the possibility of solving the problem of autonomous driving of mobile robots and improving their performance with the application of the HER technique. In addition, this result can be connected to studies that elaborately design reward systems to address complex environments. Successfully implementing autonomous driving in complex environments requires a designed compensation system, which is very difficult to devise. The proposed method can contribute to reducing the burden of reward system design and improving the performance by applying HER with reward design.

However, this study has the following limitations:

- This study contributed to the existing literature by highlighting the possibility of applying HER to a reinforcement learning-based autonomous driving algorithm. However, since the environment under consideration was rather simple, future development will need to ensure that it is applicable to complex environments as well.
- This study examined the possibility of solving the sparse reward system by applying HER. Therefore, to improve the performance of autonomous driving, additional research may be required to optimize the number of HER occurrences and devise a method of selecting an arbitrary destination.
- In this study, autonomous driving was implemented with the consideration that the size of the simulation environment differed from the size of the actual experimental environment in the realization process; however, the strategy may not operate normally under conditions different from the learning environment.
- For the proposed technique, a sudden decline in performance after a certain period following the identification of the optimal policy is common; therefore, terminating the learning process or fundamentally analyzing and improving the problem after determining the optimal policy is necessary.

In future research, we plan to verify the validity of the proposed method in a complex environment. Subsequently, studies will be conducted on autonomous driving in complex environments with static or dynamic obstacles by applying the reward system design and proposed method. In this process, additional research will be conducted on the application method of HER, as well as on overcoming the fourth limitation.

## 7. Conclusions

In this study, we developed a technique for applying HER to overcome the problems attributed to sparse reward environments in the autonomous driving of mobile robots based on the reinforcement learning algorithm, DDPG. The proposed method revealed that applying the HER technique helped overcome learning failures caused by the sparse reward problem that occurs during the autonomous driving of mobile robots by generating three normal episodes from failed episodes. The validity of the proposed method was demonstrated through two successful experiments using the reward system and in a real environment. Therefore, the contribution of this study is that the results demonstrate that the HER method can be employed for autonomous driving problems.

## Data Availability

Not applicable.

## Abbreviations

The following abbreviations are used in this manuscript:

DDPG	Deep deterministic policy gradient
HER	Hindsight experience replay
SLAM	Simultaneous localization and mapping
DQN	Deep Q network
DPG	Deterministic policy gradient
OU	Ornstein–Uhlenbeck
ROS	Robot operating system
LDS	Laser distance sensor
MLP	Multilayer perceptron
ReLU	Rectified linear unit
